# Antitumor Activities of Chimeric Anti-EphA2 Antibodies in Xenograft Models of Breast, Pancreatic, and Colorectal Cancers

**DOI:** 10.3390/ijms27073221

**Published:** 2026-04-02

**Authors:** Guanjie Li, Hiroyuki Suzuki, Tomokazu Ohishi, Hiroyuki Satofuka, Kenichiro Ishikawa, Kai Shimizu, Airi Nomura, Haruto Araki, Naoki Kojo, Kaito Suzuki, Saori Handa, Takuro Nakamura, Miyuki Yanaka, Tomohiro Tanaka, Mika K. Kaneko, Yukinari Kato

**Affiliations:** 1 Department of Antibody Drug Development, Tohoku University Graduate School of Medicine, 2-1 Seiryo-machi, Aoba-ku, Sendai 980-8575, Japan; guanjie.li.e3@tohoku.ac.jp (G.L.); hiroyuki.satofuka.e1@tohoku.ac.jp (H.S.); ken.ishikawa.r3@dc.tohoku.ac.jp (K.I.); shimizu.kai.p1@dc.tohoku.ac.jp (K.S.); nomura.airi.p3@dc.tohoku.ac.jp (A.N.); araki.haruto.r8@dc.tohoku.ac.jp (H.A.); kojo.naoki.q4@dc.tohoku.ac.jp (N.K.); suzuki.kaito.q1@dc.tohoku.ac.jp (K.S.); saori.handa.d3@tohoku.ac.jp (S.H.); takuro.nakamura.a2@tohoku.ac.jp (T.N.); miyuki.yanaka.c5@tohoku.ac.jp (M.Y.); tomohiro.tanaka.b5@tohoku.ac.jp (T.T.); mika.kaneko.d4@tohoku.ac.jp (M.K.K.); 2 Institute of Microbial Chemistry (BIKAKEN), Laboratory of Oncology, Microbial Chemistry Research Foundation, 3-14-23 Kamiosaki, Shinagawa-ku, Tokyo 141-0021, Japan; ohishit@bikaken.or.jp

**Keywords:** monoclonal antibody therapy, EphA2, ADCC, CDC, breast cancer, pancreatic cancer, colorectal cancer

## Abstract

Erythropoietin-producing hepatocellular receptor A2 (EphA2) has emerged as a key mediator that promotes tumor malignant progression. EphA2 overexpression and its non-canonical signaling lead to oncogenic transformation, metabolic reprogramming, resistance to treatments, and metastasis. Therefore, strategies targeting EphA2 have been evaluated in clinical trials. However, the clinical effects were not sufficient. An anti-EphA2 monoclonal antibody (mAb), Ea_2_Mab-7 (mouse IgG_1_, κ), demonstrated high affinity and specificity among Eph receptors. In this study, we produced recombinant class-switched Ea_2_Mab-7 variants, including Ea_2_Mab-7-mG_2a_ (mouse IgG_2a_) and Ea_2_Mab-7-hG_1_ (human IgG_1_). Both Ea_2_Mab-7-mG_2a_ and Ea_2_Mab-7-hG_1_ recognized human triple-negative breast cancer MDA-MB-231, pancreatic cancer MIA PaCa-2, and colorectal cancer HCT-15 in flow cytometry. Furthermore, both Ea_2_Mab-7-mG_2a_ and Ea_2_Mab-7-hG_1_ exerted significant antibody-dependent cellular cytotoxicity and complement-dependent cytotoxicity against these tumors. In mouse xenograft models of breast, pancreatic, and colorectal cancers, both mAbs demonstrated antitumor activity. These results indicate the potential of Ea_2_Mab-7 variants for the treatment of EphA2-positive cancers.

## 1. Introduction

The erythropoietin-producing hepatocellular (Eph) receptor family constitutes the largest subclass of receptor tyrosine kinases (RTKs) [1] and orchestrates essential physiological processes including embryonic patterning, axon guidance, synaptic formation, and vascular remodeling [2,3,4]. Eph receptors are also implicated in numerous pathological conditions, including cardiovascular diseases [5], disorders of the central nervous system [6], viral infections [7], and various tumors [1]. Among the 14 Eph receptors, EphA2 has been extensively studied in multiple solid tumors [1]. Notably, its functional role remains context-dependent and sometimes paradoxical, as EphA2 signaling can exert either pro-tumorigenic or anti-tumorigenic effects depending on ligand availability [8] and cellular or tumor-specific settings [9].

EphA2 binds to ephrin-A ligands, which facilitate intercellular communication between identical or distinct cell types through bidirectional signaling, forward and reverse signaling [4,10,11]. This bidirectional signaling of the EphA2–ephrin-A complex is further amplified by the formation of higher-order oligomeric assemblies, referred to as receptor–ligand clustering [1]. The canonical EphA2 forward signaling is mediated by its RTK activity, which is linked to tumor-suppressing activities [3]. In contrast, non-canonical EphA2 signaling, which is independent of ligand engagement and RTK activity, is characterized by phosphorylation of S897 in the short segment of intracellular domain [8,12]. The S897 is phosphorylated by AKT [8], ribosomal S6 kinase [13], and protein kinase A families [14], which have been widely associated with tumor-promoting activities. EphA2 overexpression and the non-canonical signaling involving S897 phosphorylation lead to oncogenic transformation [15], metabolic reprogramming [16], resistance to treatments [17,18,19], and metastasis [20]. The EphA2 overexpression and/or the S897 phosphorylation are closely associated with poor clinical outcomes in breast cancer [21], pancreatic cancer [22], and colorectal cancer [23]. Therefore, EphA2 has emerged as a promising therapeutic target by various modalities, including monoclonal antibody (mAb) [1].

Several EphA2–targeting mAbs have been generated that either activate or inhibit forward signaling. They promote receptor internalization, elicit antibody-dependent cellular cytotoxicity (ADCC), or serve as delivery vehicles for antitumor agents and imaging probes to EphA2-positive tumors [1]. Among EphA2-directed mAbs showing preclinical efficacy are MM-310, an antagonistic antibody used to direct liposomes containing a docetaxel prodrug to tumor sites [24]; MEDI-547, an antibody–drug conjugate (ADC) linked to monomethyl auristatin F [25]; and DS-8895a, an afucosylated mAb to enhance ADCC [26]. However, clinical development of MM-310, MEDI-547, and DS-8895a was terminated due to unacceptable toxicities—bleeding, coagulation abnormalities and elevations in liver enzymes in MEDI-547 [27], peripheral neuropathy known as a docetaxel-associated toxicity in MM-310 [1,28], and limited therapeutic efficacy in DS-8895a [29].

To target EphA2 receptors, our group has developed mAbs against EphA2 (Ea_2_Mabs) using the Cell-Based Immunization and Screening (CBIS) method. Among 94 clones of Ea_2_Mabs, an anti-EphA2 mAb (clone Ea_2_Mab-7) detects EphA2-positive cells in flow cytometry, Western blotting, and immunohistochemistry [30]. In this study, we engineered Ea_2_Mab-7 into a mouse IgG_2a_-type (Ea_2_Mab-7-mG_2a_) and a human IgG_1_-type (Ea_2_Mab-7-hG_1_) mAb and evaluated ADCC, complement-dependent cytotoxicity (CDC), and antitumor efficacy in EphA2-positive tumor xenograft models.

## 2. Results

### 2.1. Production of Class-Switched mAbs from Ea_2_Mab-7

**Figure 1:**
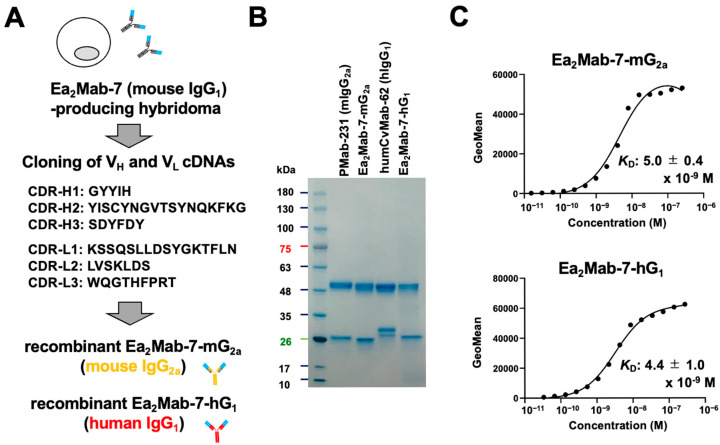
Production of Ea_2_Mab-7-mG_2a_ and Ea_2_Mab-7-hG_1_. (**A**) After determination of CDRs of Ea_2_Mab-7 (mouse IgG_1_), recombinant Ea_2_Mab-7-mG_2a_ (mouse IgG_2a_) and Ea_2_Mab-7-hG_1_ (human IgG_1_) were produced. The amino acid sequences of V_H_ and V_L_ CDRs were indicated. (**B**) PMab-231 (control mIgG_2a_), Ea_2_Mab-7-mG_2a_, humCvMab-62 (control hIgG_1_), and Ea_2_Mab-7-hG_1_ were subject to SDS-PAGE, and the gel was stained with Bio-Safe CBB G-250 Stain. (**C**) Determination of the binding affinity of Ea_2_Mab-7-mG_2a_ and Ea_2_Mab-7-hG_1_ using flow cytometry. CHO/EphA2 was suspended in Ea_2_Mab-7-mG_2a_ and Ea_2_Mab-7-hG_1_ at indicated concentrations, followed by Alexa Fluor 488-conjugated anti-mouse IgG treatment. The SA3800 Cell Analyzer (Sony Corp., Tokyo, Japan) was used to analyze fluorescence data. The average *K*_D_ values (±standard deviation) from three independent measurements were calculated by GraphPad PRISM 6 software. The representative graphs were shown.

We previously reported that Ea_2_Mab-7, an anti-EphA2 mAb, detects EphA2-positive cells by flow cytometry, Western blotting, and immunohistochemistry [30]. We first confirmed the specificity of Ea_2_Mab-7 using nine EphA and five EphB receptor-overexpressed CHO-K1 [31]. As shown in supplementary Figure S1, Ea_2_Mab-7 showed the binding to CHO/EphA2, with no or very little cross-reactivity to other Eph receptors observed at 10 μg/mL. We next determined the V_H_ and V_L_ complementarity-determining region (CDR) sequences of Ea_2_Mab-7 (Figure 1A). Furthermore, we engineered a mouse IgG_2a_-type Ea_2_Mab-7 (Ea_2_Mab-7-mG_2a_) and human IgG_1_-type Ea_2_Mab-7 (Ea_2_Mab-7-hG_1_) by fusing the V_H_ and V_L_ of Ea_2_Mab-7 with the C_H_ and C_L_ chains of mouse IgG_2a_ and human IgG_1_, respectively (Figure 1A). Isotype control mAbs, PMab-231 (mouse IgG_2a_, referred to as control mIgG_2a_) and humCvMab-62 (human IgG_1,_ referred to as control hIgG_1_) were also prepared. Under reduced conditions, we confirmed the purity of original and recombinant mAbs by SDS-PAGE (Figure 1B).

Next, the binding affinity was investigated using flow cytometry. The dissociation constant (*K*_D_) values of Ea_2_Mab-7-mG_2a_ and Ea_2_Mab-7-hG_1_ for CHO/EphA2 were determined to be (5.0 ± 0.4) × 10^−9^ M and (4.4 ± 1.0) × 10^−9^ M, respectively (Figure 1C). These results indicated that Ea_2_Mab-7-mG_2a_ and Ea_2_Mab-7-hG_1_ retain the affinity with parental mAb, Ea_2_Mab-7 as reported previously (*K*_D_: 6.2 × 10^−9^ M) [30].

### 2.2. Flow Cytometry Using Ea_2_Mab-7-mG_2a_ and Ea_2_Mab-7-hG_1_ in EphA2-Positive Cancer Cells

**Figure 2:**
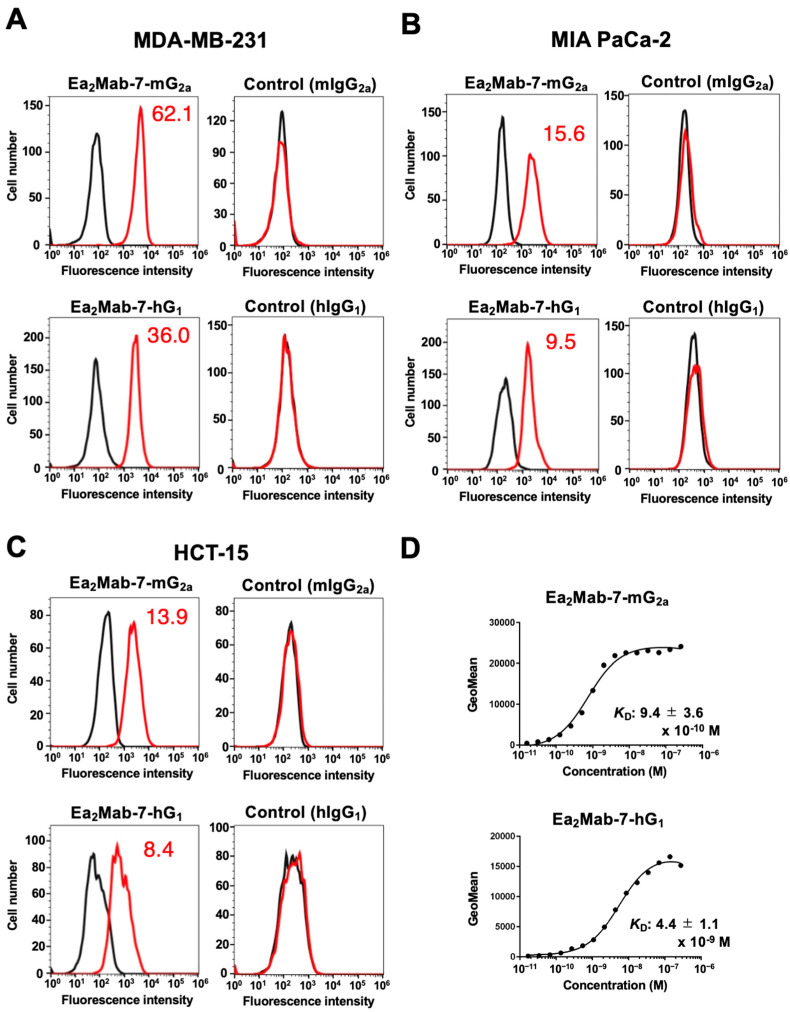
Reactivity of Ea_2_Mab-7-mG_2a_ and Ea_2_Mab-7-hG_1_ to tumor cells. (**A**–**C**) Flow cytometry using control mIgG_2a_, Ea_2_Mab-7-mG_2a_, control hIgG_1_, and Ea_2_Mab-7-hG_1_ (0.1 μg/mL; Red line) or buffer control (Black line) against breast cancer MDA-MB-231 (**A**), pancreatic cancer MIA PaCa-2 (**B**), and colorectal cancer HCT-15 (**C**). After treatment with primary mAbs, cells were treated with Alexa Fluor 488-conjugated anti-mouse IgG or fluorescein isothiocyanate (FITC)-conjugated anti-human IgG. The GeoMean ratio to buffer control was shown. (**D**) Determination of the binding affinity of Ea_2_Mab-7-mG_2a_ and Ea_2_Mab-7-hG_1_ using flow cytometry. MDA-MB-231 was suspended in Ea_2_Mab-7-mG_2a_ and Ea_2_Mab-7-hG_1_ at indicated concentrations, followed by Alexa Fluor 488-conjugated anti-mouse IgG or FITC-conjugated anti-human IgG treatment. Fluorescence data were analyzed using the SA3800 Cell Analyzer. The average *K*_D_ values (±standard deviation) from three independent measurements were calculated by GraphPad PRISM 6 software. The representative graphs were shown.

We next screened the EphA2 expression in more than 100 cell lines using flow cytometry. Among them, we chose human triple-negative breast cancer (TNBC) MDA-MB-231, pancreatic cancer MIA PaCa-2, and colorectal cancer HCT-15 based on their reactivity and availability in mouse xenograft models. As shown in Figure 2A–C, Ea_2_Mab-7-mG_2a_ recognized MDA-MB-231, MIA PaCa-2, and HCT-15 in flow cytometry at 0.1 µg/mL. In contrast, control mIgG_2a_ did not. Ea_2_Mab-7-hG_1_ also showed similar reactivity at 0.1 µg/mL, but control hIgG_1_ did not (Figure 2A–C). The dissociation constant (*K*_D_) values of Ea_2_Mab-7-mG_2a_ and Ea_2_Mab-7-hG_1_ for MDA-MB-231 were determined to be (9.4 ± 3.6) × 10^−10^ M and (4.4 ± 1.1) × 10^−9^ M, respectively (Figure 2D). These results indicated that both Ea_2_Mab-7-mG_2a_ and Ea_2_Mab-7-hG_1_ exhibit high binding affinity for MDA-MB-231.

### 2.3. ADCC and CDC Elicited by Ea_2_Mab-7-mG_2a_ Against EphA2-Positive Cancer Cells

**Figure 3:**
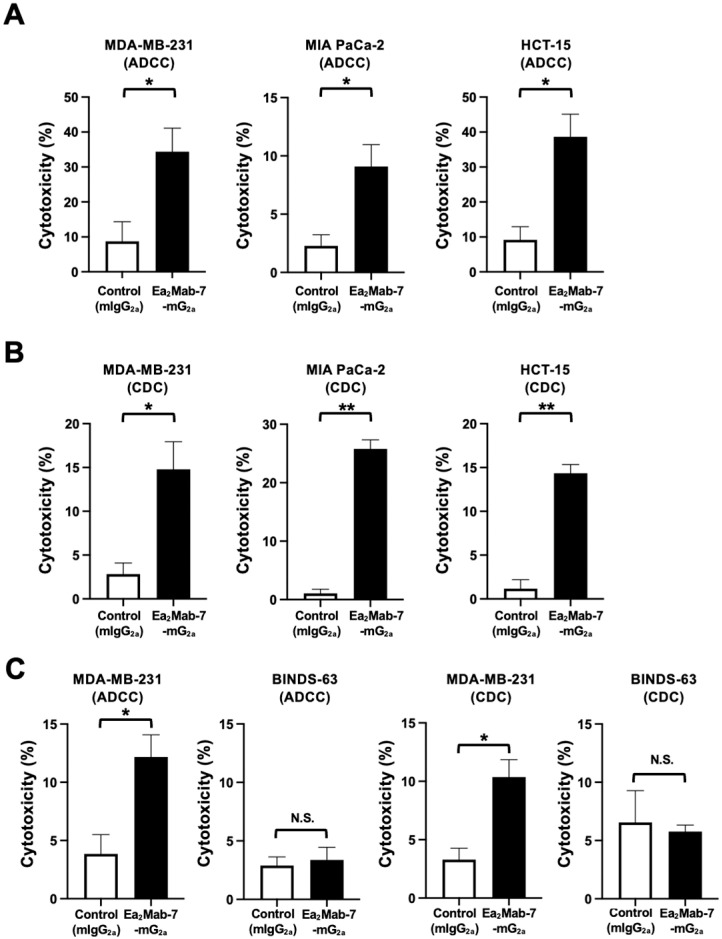
ADCC and CDC by Ea_2_Mab-7-mG_2a_ against EphA2-positive tumor cells. The target cells labeled with Calcein AM (MDA-MB-231, MIA PaCa-2, and HCT-15) were incubated with effector splenocytes derived from BALB/c nude mice (**A**) or rabbit complement (**B**) in the presence of Ea_2_Mab-7-mG_2a_ or control mIgG_2a_. (**C**) ADCC and CDC assays were performed using MDA-MB-231 and EphA2-knockout MDA-MB-231 (BINDS-63). Calcein release into the medium was measured, and cytotoxicity was determined. Values are shown as the mean ± SEM (n = 3). Asterisks indicate statistical significance (** *p* < 0.01 and * *p* < 0.05; two-tailed unpaired *t*-test). N.S., not significant.

We investigated the effect of Ea_2_Mab-7-mG_2a_ on cell proliferation in vitro. As shown in supplementary Figure S2, Ea_2_Mab-7-mG_2a_ did not affect cell proliferation in MDA-MB-231, HCT-15, and EphA2-positive normal fibroblast KMST-6. We next investigated whether Ea_2_Mab-7-mG_2a_ exhibits ADCC and CDC against EphA2-positive MDA-MB-231, MIA PaCa-2, and HCT-15. The ADCC induced by Ea_2_Mab-7-mG_2a_ and control mIgG_2a_ was investigated in the presence of effector splenocytes derived from BALB/c nude mice. As shown in Figure 3A, Ea_2_Mab-7-mG_2a_ induced potent ADCC against MDA-MB-231 (34.4% cytotoxicity; *p* < 0.05) compared to the control mIgG_2a_ (8.7% cytotoxicity). Ea_2_Mab-7-mG_2a_ elicited ADCC against MIA PaCa-2 (9.0% cytotoxicity; *p* < 0.05) more effectively than the control mIgG_2a_ (2.3% cytotoxicity). Furthermore, Ea_2_Mab-7-mG_2a_ also showed potent ADCC against HCT-15 (38.7% cytotoxicity; *p* < 0.05) more effectively than the control mIgG_2a_ (9.2% cytotoxicity).

The CDC elicited by Ea_2_Mab-7-mG_2a_ was next investigated in the presence of complements. As shown in Figure 3B, Ea_2_Mab-7-mG_2a_ elicited significant CDC against MDA-MB-231 (14.8% cytotoxicity; *p* < 0.05) compared to the control mIgG_2a_ (2.8% cytotoxicity). Ea_2_Mab-7-mG_2a_ induced potent CDC against MIA PaCa-2 (25.8% cytotoxicity; *p* < 0.01) more effectively than the control mIgG_2a_ (1.0% cytotoxicity). Additionally, Ea_2_Mab-7-mG_2a_ showed CDC against HCT-15 (14.8% cytotoxicity; *p* < 0.01) more effectively than the control mIgG_2a_ (2.8% cytotoxicity).

We also established EphA2-knockout MDA-MB-231 (BINDS-63, supplementary Figure S3) and found that the ADCC and CDC were not elicited by Ea_2_Mab-7-mG_2a_ against BINDS-63 (Figure 3C). These results indicated that Ea_2_Mab-7-mG_2a_ exerted ADCC and CDC in the presence of effector splenocytes and complements, respectively.

### 2.4. Antitumor Effects of Ea_2_Mab-7-mG_2a_ Against EphA2-Positive Cancer Cells

**Figure 4:**
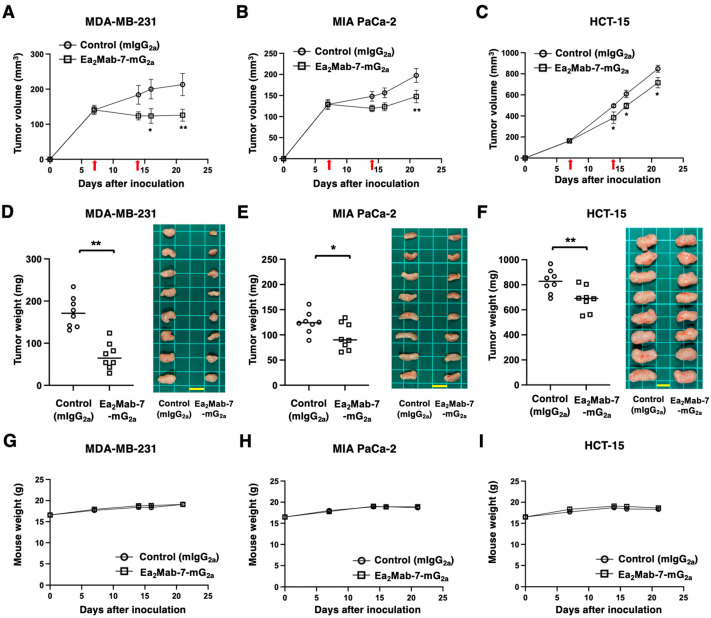
Antitumor activity of Ea_2_Mab-7-mG_2a_ against human tumor xenografts. (**A**–**C**) MDA-MB-231 (**A**), MIA PaCa-2 (**B**), and HCT-15 (**C**) were subcutaneously injected into BALB/c nude mice (day 0). Ea_2_Mab-7-mG_2a_ (100 μg) or control mIgG_2a_ (100 μg) were intraperitoneally injected into each mouse on days 7 and 14 (arrows). The tumor volume is represented as the mean ± SEM. * *p* < 0.05, ** *p* < 0.01 (two-way ANOVA with Sidak’s post hoc test). (**D**–**F**) After cell inoculation, the mice were euthanized on day 21. The tumor weights (left) and appearance (right) of MDA-MB-231 (**D**), MIA PaCa-2 (**E**), and HCT-15 (**F**) xenografts were measured. Values are presented as the mean ± SEM. ** *p* < 0.01 and * *p* < 0.05 (two-tailed unpaired *t*-test). Scale bar, 1 cm. (**G**–**I**) Body weight (mean ± SEM) of xenograft-bearing mice treated with the mAbs is presented. There is no significant difference (two-way ANOVA with Sidak’s post hoc test).

After the inoculation of MDA-MB-231, MIA PaCa-2, or HCT-15 at the left flanks of BALB/c nude mice, Ea_2_Mab-7-mG_2a_ or control mIgG_2a_ was intraperitoneally injected into the xenograft-bearing mice on days 7 and 14. The tumor volume was measured on the indicated days. The Ea_2_Mab-7-mG_2a_ administration resulted in a significant reduction in MDA-MB-231 xenografts on days 16 (*p* < 0.05) and 21 (*p* < 0.01) compared with that of control mIgG_2a_ (Figure 4A). In the MIA PaCa-2 xenograft, a significant reduction was observed on day 21 (*p* < 0.01) (Figure 4B). In the HCT-15 xenograft, a significant reduction was also observed on days 14 (*p* < 0.05), 16 (*p* < 0.05), and 21 (*p* < 0.05) (Figure 4C).

In the xenograft weight, Ea_2_Mab-7-mG_2a_ showed the reduction in MDA-MB-231 (60% reduction; *p* < 0.01; Figure 4D), MIA PaCa-2 (22% reduction; *p* < 0.05; Figure 4E), and HCT-15 (17% reduction; *p* < 0.01; Figure 4F) compared with control mIgG_2a_. The resected MDA-MB-231, MIA PaCa-2, and HCT-15 tumors on day 21 are shown in each figure. The xenograft-bearing mice did not lose body weight by Ea_2_Mab-7-mG_2a_ treatment (Figure 4G–I).

### 2.5. ADCC and CDC Elicited by Ea_2_Mab-7-hG_1_ Against EphA2-Positive Cancer Cells

**Figure 5:**
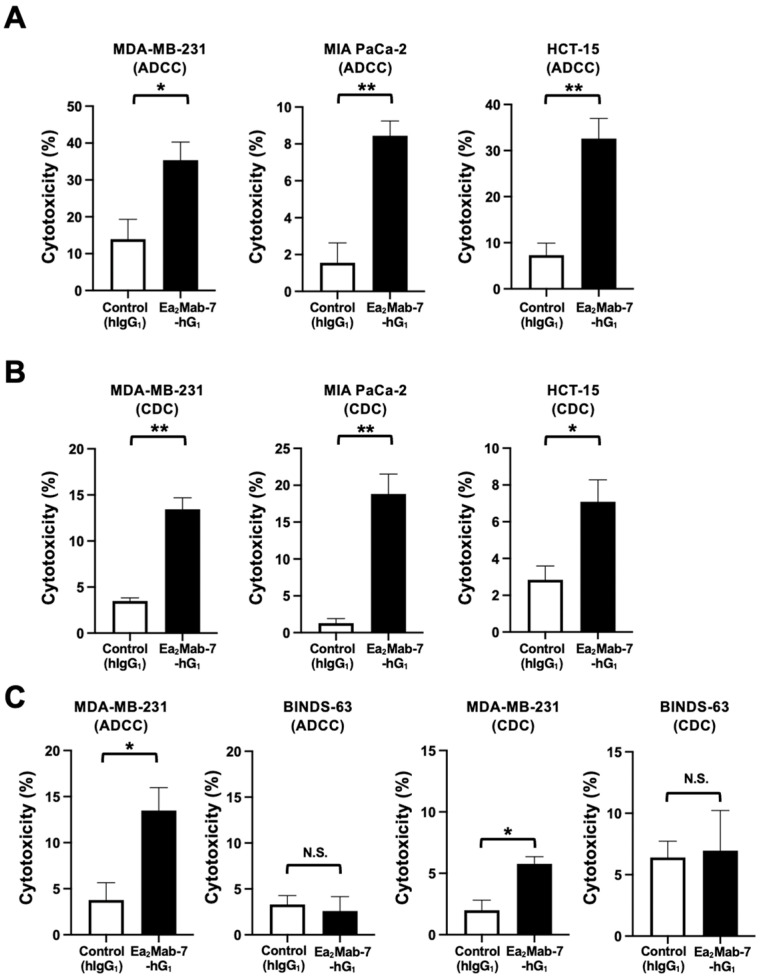
ADCC and CDC by Ea_2_Mab-7-hG_1_ against EphA2-positive tumor cells. The target cells labeled with Calcein AM (MDA-MB-231, MIA PaCa-2, and HCT-15) were incubated with effector splenocytes derived from BALB/c nude mice (**A**) or rabbit complement (**B**) in the presence of Ea_2_Mab-7-hG_1_ or control hIgG_1_. (**C**) ADCC and CDC assays were performed using MDA-MB-231 and EphA2-knockout MDA-MB-231 (BINDS-63). Calcein release into the medium was measured, and cytotoxicity was determined. Values are shown as the mean ± SEM (n = 3). Asterisks indicate statistical significance (** *p* < 0.01 and * *p* < 0.05; two-tailed unpaired *t*-test). N.S., not significant.

We next investigated whether Ea_2_Mab-7-hG_1_ exhibits ADCC and CDC against EphA2-positive MDA-MB-231, MIA PaCa-2, and HCT-15. We also used the BALB/c nude mice-derived splenocytes as effector cells because all four mouse Fcγ receptors bind to human IgG_1_, which induces ADCC in the presence of mouse effector cells [32]. Therefore, the ADCC induced by Ea_2_Mab-7-hG_1_ and control hIgG_1_ was investigated in the presence of BALB/c nude mice-derived splenocytes. We first confirmed that ADCC and CDC against MDA-MB-231 were elicited by a similar dose of Ea_2_Mab-7-hG_1_ compared to Ea_2_Mab-7-mG_2a_ (supplementary Figure S4). As shown in Figure 5A, Ea_2_Mab-7-hG_1_ induced potent ADCC against MDA-MB-231 (35.3% cytotoxicity; *p* < 0.05) compared to the control hIgG_1_ (14.0% cytotoxicity). Ea_2_Mab-7-hG_1_ elicited ADCC against MIA PaCa-2 (8.4% cytotoxicity; *p* < 0.01) more effectively than the control hIgG_1_ (1.6% cytotoxicity). Furthermore, Ea_2_Mab-7-hG_1_ also showed potent ADCC against HCT-15 (32.6% cytotoxicity; *p* < 0.01) more effectively than the control hIgG_1_ (7.3% cytotoxicity).

The CDC elicited by Ea_2_Mab-7-hG_1_ was next investigated in the presence of complements. As shown in Figure 5B, Ea_2_Mab-7-hG_1_ elicited significant CDC against MDA-MB-231 (13.4% cytotoxicity; *p* < 0.01) compared to the control hIgG_1_ (3.5% cytotoxicity). Ea_2_Mab-7-hG_1_ induced potent CDC against MIA PaCa-2 (18.8% cytotoxicity; *p* < 0.01) more effectively than the control hIgG_1_ (1.3% cytotoxicity). Additionally, Ea_2_Mab-7-hG_1_ showed CDC against HCT-15 (7.1% cytotoxicity; *p* < 0.05) more effectively than the control hIgG_1_ (2.8% cytotoxicity).

The ADCC and CDC were not elicited by Ea_2_Mab-7-hG_1_ against BINDS-63 (Figure 5C). These results indicated that Ea_2_Mab-7-hG_1_ exerted ADCC and CDC in the presence of effector splenocytes and complements, respectively.

### 2.6. Antitumor Effects of Ea_2_Mab-7-hG_1_ Against EphA2-Positive Cancer Cells

**Figure 6:**
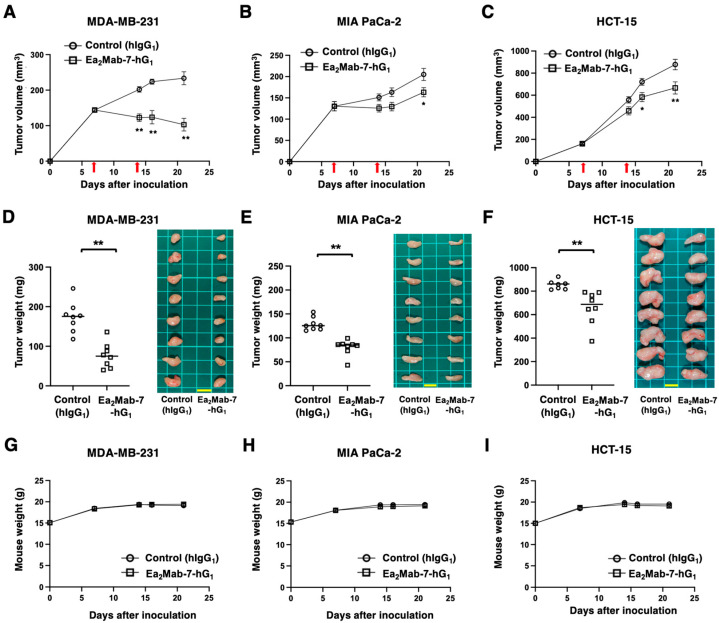
Antitumor activity of Ea_2_Mab-7-hG_1_ against human tumor xenografts. (**A**–**C**) MDA-MB-231 (**A**), MIA PaCa-2 (**B**), and HCT-15 (**C**) cells were subcutaneously injected into BALB/c nude mice (day 0). Ea_2_Mab-7-hG_1_ (100 μg) or control hIgG_1_ (100 μg) were intraperitoneally injected into each mouse on days 7 and 14 (arrows). The tumor volume is represented as the mean ± SEM. * *p* < 0.05, ** *p* < 0.01 (two-way ANOVA with Sidak’s post hoc test). (**D**–**F**) After cell inoculation, the mice were euthanized on day 21. The tumor weights (left) and appearance (right) of MDA-MB-231 (**D**), MIA PaCa-2 (**E**), and HCT-15 (**F**) xenografts were measured. Values are presented as the mean ± SEM. ** *p* < 0.01 (two-tailed unpaired *t*-test). Scale bar, 1 cm. (**G**–**I**) Body weight (mean ± SEM) of xenograft-bearing mice treated with the mAbs is presented. There is no significant difference (two-way ANOVA with Sidak’s post hoc test).

In preclinical studies of clinically approved mAbs, such as trastuzumab (human IgG_1_), the antitumor efficacy was demonstrated in nude mice bearing breast cancer xenografts in the absence of human-derived effectors [33,34,35]. Therefore, the antitumor effect of Ea_2_Mab-7-hG_1_ was examined in cancer xenografts inoculated in nude mice. After the inoculation of MDA-MB-231, MIA PaCa-2, or HCT-15 at the left flanks of BALB/c nude mice, Ea_2_Mab-7-hG_1_ or control hIgG_1_ was intraperitoneally injected into the xenograft-bearing mice on days 7 and 14. The tumor volume was measured on the indicated days. The Ea_2_Mab-7-hG_1_ administration resulted in a potent reduction in MDA-MB-231 xenografts on days 14 (*p* < 0.01), 16 (*p* < 0.01), and 21 (*p* < 0.01) compared with that of control hIgG_1_ (Figure 6A). In the MIA PaCa-2 xenograft, a significant reduction was observed on day 21 (*p* < 0.05) (Figure 6B). In the HCT-15 xenograft, a significant reduction was also observed on days 16 (*p* < 0.05) and 21 (*p* < 0.01) (Figure 6C).

In the xenograft weight, Ea_2_Mab-7-hG_1_ showed the reduction in MDA-MB-231 (56% reduction; *p* < 0.01; Figure 6D), MIA PaCa-2 (38% reduction; *p* < 0.01; Figure 6E), and HCT-15 (26% reduction; *p* < 0.01; Figure 6F) compared with control hIgG_1_. The resected MDA-MB-231, MIA PaCa-2, and HCT-15 tumors on day 21 are shown in each figure. The xenograft-bearing mice did not lose body weight by Ea_2_Mab-7-hG_1_ treatment (Figure 6G–I).

## 3. Discussion

This study demonstrated the in vitro and in vivo antitumor efficacy of a novel mAb against EphA2. Both Ea_2_Mab-7-mG_2a_ and Ea_2_Mab-7-hG_1_ recognized MDA-MB-231, MIA PaCa-2, and HCT-15 in flow cytometry (Figure 2). We compared the ADCC, CDC (Figure 3 and Figure 5), and in vivo antitumor effects (Figure 4 and Figure 6) between Ea_2_Mab-7-mG_2a_ and Ea_2_Mab-7-hG_1_ in the same experimental setting. Although the binding affinity to MDA-MB-231 differed between Ea_2_Mab-7-mG_2a_ and Ea_2_Mab-7-hG_1_ (Figure 2D), the in vitro and in vivo efficacy was similar, suggesting that Ea_2_Mab-7-hG_1_ activated the effectors and exhibited antitumor efficacy in mice. In Figure 2, we quantified EphA2 expressions in MDA-MB-231, MIA PaCa-2, and HCT-15. The expression levels were correlated with the in vivo antitumor efficacy (Figure 4 and Figure 6), suggesting that cell-surface expression of EphA2 influences antitumor efficacy by Ea_2_Mab-7-mG_2a_ or Ea_2_Mab-7-hG_1_. Additionally, we have not evaluated the antitumor effect of Ea_2_Mab-7 derivatives in immunocompetent models. Therefore, mouse syngeneic tumor models should be investigated if Ea_2_Mab-7 sufficiently recognizes mouse EphA2.

DS-8895a, an afucosylated anti-EphA2 mAb, was previously developed to potentiate ADCC [26]. However, the clinical trial of DS-8895a was terminated due to low tumor uptake and therapeutic efficacy in advanced solid tumor patients [29,36]. Therefore, selecting tumor samples and/or measuring EphA2 abundance are thought to be essential. Since Ea_2_Mab-7 is suitable for detecting EphA2 in immunohistochemistry using a clinically approved staining system [30], the validation of EphA2 expression by Ea_2_Mab-7 is necessary in future studies.

In metastatic colorectal cancer, EphA2 levels are significantly associated with worse outcome in patients treated with FOLFIRI plus cetuximab, an anti-EGFR mAb [37]. In HCT-15 xenograft models, ALW-II-41-27 (an EphA2 tyrosine kinase inhibitor) and cetuximab synergistically inhibited the xenograft growth [37]. Therefore, the combined effect of Ea_2_Mab-7-mG_2a_ or Ea_2_Mab-7-hG_1_ with cetuximab should be evaluated in preclinical models.

In pancreatic ductal adenocarcinoma (PDAC), EphA2 was identified as a candidate tumor-intrinsic driver of immunosuppression [38]. EphA2 expression in PDAC promotes an immunosuppressive tumor microenvironment that confers resistance to combination immunotherapy [38]. Since the EphA2-prostaglandin endoperoxide synthase 2 (PTGS2) axis mediated the T cell exclusion and was associated with poor patient survival, the blockade by anti-EphA2 mAbs may represent a therapeutic strategy for immunotherapy-resistant PDAC. Ea_2_Mab-7-mG_2a_ or Ea_2_Mab-7-hG_1_ should be evaluated to determine whether they possess the inhibitory effect of the EphA2-PTGS2 axis and evaluate the antitumor effect in combination with immunotherapy.

The oncogenic herpesviruses such as Epstein–Barr virus (EBV) persistently infect over 90% of adults worldwide, leading to the development of B cell or epithelial malignancies [39]. EBV is responsible for approximately 2% of all cancers, including lymphomas (e.g., Burkitt’s lymphoma and Hodgkin’s lymphoma) and carcinomas (e.g., nasopharyngeal, gastric, and breast cancers) [40,41]. Studies have reported that EBV-associated carcinomas arise from clonal expansion of infected cells, suggesting that early infection events are sufficient to initiate carcinogenesis [42]. EBV utilizes different viral glycoproteins and distinct host receptors to infect human B cells and epithelial cells [43]. EBV entry into an epithelial cell involves different attachment and fusion proteins mediated by interactions between viral glycoproteins (gH/gL, gB) and host receptors, EphA2 [44] and R9AP (RGS9-1 anchor protein) [45]. In epithelial cells, gH/gL simultaneously binds to EphA2 and R9AP, which leads to gB-mediated viral and host membrane fusion [45]. Ganoderma microsporum immunomodulatory protein interacts with the gB and the host epithelial receptor EphA2, which disrupt viral and host membrane fusion [46]. Although the neutralizing effect of anti-EphA2 mAbs on EBV infection has not been evaluated, anti-EphA2 mAbs, including Ea_2_Mab-7-mG_2a_ and Ea_2_Mab-7-hG_1_, have the potential to control the infection and tumorigenesis in EBV-associated carcinomas.

A clinical trial of MEDI-547, an anti-EphA2 mAb-ADC, was discontinued due to the unacceptable toxicities such as bleeding, coagulation abnormalities, and elevations in liver enzymes [27]. Since EphA2 is expressed in various types of normal cells, these anti-EphA2 mAbs may recognize normal cells. As shown in supplementary Figure S5, Ea_2_Mab-7 recognized normal fibroblast (KMST-6) and epithelial cell lines (293FT and hTCEpi) in flow cytometry. Because Ea_2_Mab-7 similarly recognized a normal cornea epithelial cell line, hTCEpi compared to MDA-MB-231, this raises a concern about applying the modalities to clinical studies. In ADC treatment, ocular surface adverse events including dry eye, blurred vision, keratitis/keratopathy, conjunctivitis, and corneal pseudomicrocysts have been reported [47,48]. Therefore, cancer-specific mAbs (CasMabs) for EphA2 should be selected to achieve an acceptable therapeutic window with low on-target toxicity.

Our group has developed CasMabs for various antigens, including podoplanin, podocalyxin, and human epidermal growth factor receptor 2 (HER2), and has clarified the cancer-specific epitopes. In the anti-HER2 CasMab development, we selected an anti-HER2 CasMab, H_2_CasMab-2 (also known as H_2_Mab-250) from about three hundred anti-HER2 mAb clones [49]. H_2_CasMab-2 recognized HER2 in breast cancer cells but not in regular epithelial cell lines derived from the mammary gland, colon, kidney proximal tubule, and lung bronchus [49]. The epitope analyses identified a critical amino acid (Trp614) in the HER2 extracellular domain for H_2_CasMab-2 recognition [49] and solved the structure of cancer-specific recognition [50]. Furthermore, a single-chain variable fragment of H_2_CasMab-2 was developed to chimeric antigen receptor (CAR)-T cell therapy, which exhibited cancer-specific reactivity and antitumor efficacy [50]. The H_2_CasMab-2 CAR-T is currently being evaluated in a phase I clinical trial for patients with HER2-positive advanced solid tumors (NCT06241456) [50]. Therefore, selecting CasMab against EphA2 and identifying the cancer-specific epitopes are essential strategies for developing therapeutic mAbs and modalities. We have developed about 100 clones of Ea_2_Mabs and will screen for cancer-specific reactivity. Ea_2_Mab-7-mG_2a_ and Ea_2_Mab-7-hG_1_ would serve as reference mAbs to compare the antitumor efficacy with anti-EphA2 CasMabs.

## 4. Materials and Methods

### 4.1. Cell Lines

The human pancreatic cancer cell line MIA PaCa-2, the colorectal cancer cell line HCT-15, and the human embryonic fibroblast cell line KMST-6 were obtained from the Cell Resource Center for Biomedical Research, Institute of Development, Aging and Cancer, Tohoku University (Miyagi, Japan). The human TNBC MDA-MB-231 was obtained from the American Type Culture Collection (Manassas, VA, USA). Human embryonic kidney 293FT was purchased from Thermo Fisher Scientific Inc. (Thermo, Waltham, MA, USA). A TERT-expressed normal cornea epithelial cell line, hTCEpi, was purchased from EVERCYTE (Vienna, Austria). Eph receptor overexpressed Chinese hamster ovary-K1 (e.g., CHO/EphA2) were previously established [31].

EphA2-knockout MDA-MB-231 (BINDS-63) was generated using the CRISPR/Cas9 system. An EphA2-specific sgRNA targeting the sequence 5′-GCGGCGGCGCAGGGCAAGGA-3′ (TrueGuide™ Synthetic sgRNA, Thermo Fisher Scientific, Inc.) was synthesized and cloned into the GeneArt™ CRISPR Nuclease OFP Vector (Thermo Fisher Scientific, Inc.). These cell lines were cultured as described previously [30,49].

For cell proliferation assay, cells were plated in 96-well plates at a density of 1 × 10^3^ cells/well and treated with mAbs at 0.1~30 µg/mL for 6 days. The cells were treated with CellTiter-Glo^®^3D Cell Viability Assay reagent (Promega, Madison, WI, USA), and the luminescence was measured using GloMax^®^ (Promega). Cell viability (% of control) was determined.

### 4.2. Recombinant mAb Production

To generate recombinant mouse IgG_2a_-type Ea_2_Mab-7 (Ea_2_Mab-7-mG_2a_) and human IgG_1_-type Ea_2_Mab-7 (Ea_2_Mab-7-hG_1_), the V_H_ and V_L_ cDNAs of Ea_2_Mab-7 (mouse IgG_1_, κ) were cloned into pCAG-Neo and pCAG-Ble vectors (FUJIFILM Wako Pure Chemical Corporation, Osaka, Japan), together with the corresponding constant regions of mouse IgG_2a_ [51] and human IgG_1_ [52], respectively. Antibody expression vectors were transfected into ExpiCHO-S using the ExpiCHO Expression System to produce Ea_2_Mab-7-mG_2a_ and Ea_2_Mab-7-hG_1_. PMab-231 (mouse IgG_2a_) [51] and humCvMab-62 (human IgG_1_) [52] were used as isotype control human IgG_1_ (hIgG_1_) and mouse IgG_2a_ (mIgG_2a_), respectively. All antibodies were purified using Ab-Capcher (ProteNova Co., Ltd., Kagawa, Japan). These mAbs were denatured by SDS sample buffer (Nacalai Tesque, Inc., Kyoto, Japan) containing 2-mercaptoethanol and subject to SDS-PAGE. The gel was stained with Bio-Safe CBB G-250 Stain (Bio-Rad Laboratories, Inc., Berkeley, CA, USA).

### 4.3. Animals

The animal study for the antitumor efficacy of Ea_2_Mab-7-mG_2a_ and Ea_2_Mab-7-hG_1_ was approved by the Institutional Committee for Experiments of the Institute of Microbial Chemistry (approval no. 2025-040) within which the work was undertaken and that it conforms to the provisions of the Declaration of Helsinki. Humane objectives for euthanasia were established as a loss of original body weight to a point > 25% and/or a maximal tumor size > 3000 mm^3^.

### 4.4. Flow Cytometry

Cells were harvested using 1 mM ethylenediaminetetraacetic acid (EDTA; Nacalai Tesque, Inc., Kyoto, Japan) in phosphate-buffered saline (PBS). The cells were treated with primary mAbs in blocking buffer (0.1% bovine serum albumin in PBS) for 30 min at 4 °C. Then, the cells were treated with Alexa Fluor 488-conjugated anti-mouse IgG (1:2000; Cell Signaling Technology, Inc., Danvers, MA, USA) or fluorescein isothiocyanate (FITC)-conjugated anti-human IgG (1:2000; Sigma-Aldrich Corp., St. Louis, MO, USA) for 30 min at 4 °C. Fluorescence data were collected using the SA3800 Cell Analyzer (Sony Corp., Tokyo, Japan) and analyzed with FlowJo software version 10.9.0 (BD Biosciences, Franklin Lakes, NJ, USA).

### 4.5. ADCC

Five-week-old female BALB/c nude mice were purchased from Japan SLC, Inc. (Shizuoka, Japan). Effector cells were isolated from the spleens as described previously [53]. Target cells (MDA-MB-231, MIA PaCa-2, and HCT-15) were labeled with 10 µg/mL of Calcein AM (Thermo). The target cells were plated in 96-well plates at a density of 5 × 10^3^ cells/well and combined with effector cells (effector-to-target ratio, 50:1) and 100 μg/mL of either control mIgG_2a_ or Ea_2_Mab-7-mG_2a_, either control hIgG_1_ or Ea_2_Mab-7-hG_1_. After incubating for 4.5 h at 37 °C, the calcein released into the supernatant was measured as described previously [54].

### 4.6. CDC

The target cells labeled with Calcein AM (MDA-MB-231, MIA PaCa-2, and HCT-15) were seeded and combined with rabbit complement (final concentration 10%, Low-Tox-M Rabbit Complement; Cedarlane Laboratories, Hornby, ON, Canada) along with 100 μg/mL of either control mIgG_2a_ or Ea_2_Mab-7-mG_2a_, either control hIgG_1_ or Ea_2_Mab-7-hG_1_. After a 4.5 h incubation at 37 °C, the amount of calcein released into the medium was measured as described previously [54].

### 4.7. Antitumor Activities in Xenografts of Human Tumors

MDA-MB-231, MIA PaCa-2, and HCT-15 were mixed with Matrigel Matrix Growth Factor Reduced (BD Biosciences). Subcutaneous injections (5 × 10^6^ cells/mouse) were then given to the left flanks of BALB/c nude mice. On the seventh post-inoculation day, 100 µg of control mIgG_2a_ (n = 8), Ea_2_Mab-7-mG_2a_ (n = 8), control hIgG_1_ (n = 8), or Ea_2_Mab-7-hG_1_ (n = 8) in 100 µL PBS were administered intraperitoneally. Additional antibody injections were given on day 14. The tumor diameter was assessed on days 7, 14, 16, and 21 after the tumor cell implantation. Tumor volume was calculated using the formula, volume = W^2^ × L/2, where W represents the short diameter and L the long diameter. The mice’s weight was also assessed on days 7, 14, 16, and 21 following tumor cell inoculation. When observations on day 21 were complete, the mice were sacrificed, and tumor weights were assessed after tumor excision.

### 4.8. Statistical Analyses

The mean ± standard error of the mean (SEM) is presented in all data. A two-tailed unpaired *t*-test was conducted to measure ADCC, CDC, and tumor weight. ANOVA with Sidak’s post hoc test was performed for tumor volume and mouse weight. GraphPad Prism 10 (GraphPad Software, Inc., La Jolla, CA, USA) was used for all calculations. *p* < 0.05 was considered statistically significant.

## Data Availability

The data presented in this study are available in the article.

## Supplementary Materials

The following supporting information can be downloaded at https://www.mdpi.com/article/10.3390/ijms27073221/s1.
