# Supplementary material for: Antitumor Activities of Chimeric Anti-EphA2 Antibodies in Xenograft Models of Breast, Pancreatic, and Colorectal Cancers
Source: Int J Mol Sci. 2026 Apr 2;27(7):3221. doi: 10.3390/ijms27073221 (PMC13072770; doi:10.3390/ijms27073221)
Supplement: Supplementary file 1 [file ijms-27-03221-s001.zip › ijms-4165400-supplementary.pdf]

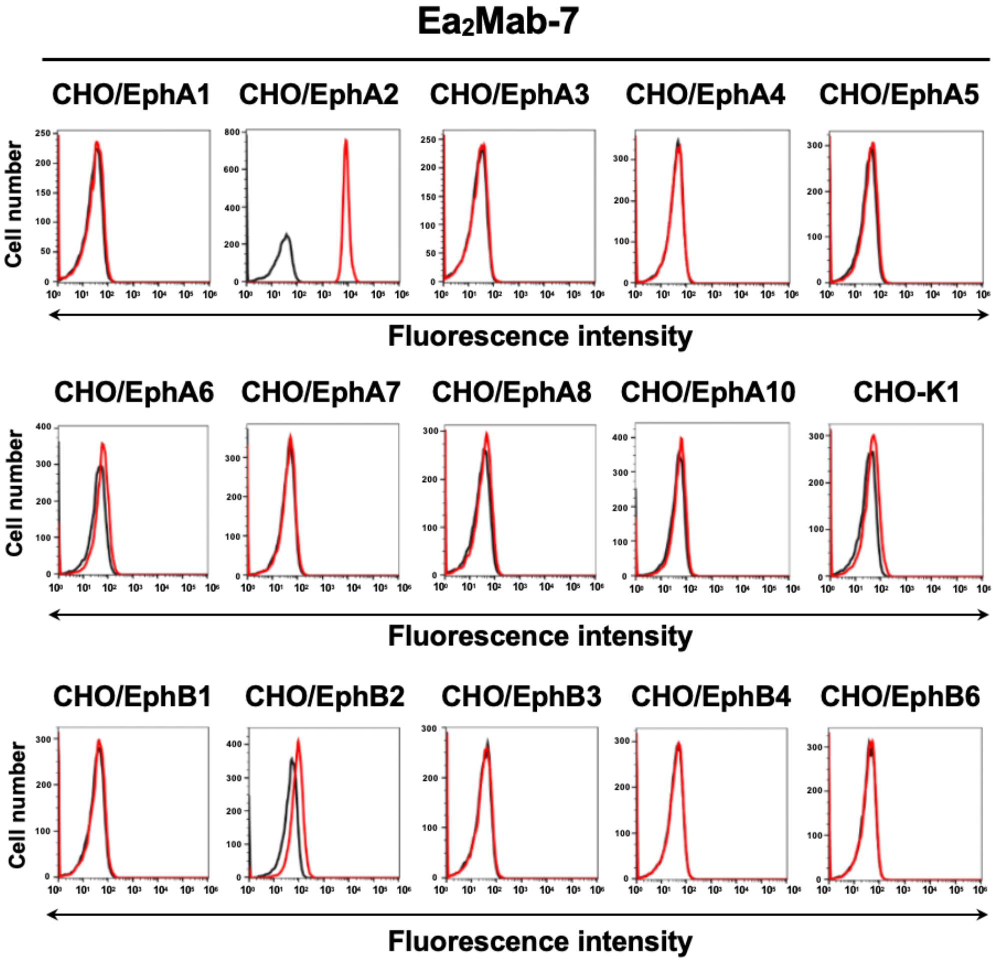

Supplementary figure S1: Cross-reactivity of Ea2Mab-7 to Eph receptor-overexpressed CHO-K1. The 14 Eph receptor-overexpressed CHO-K1 and parental CHO-K1 were treated with 10 µg/mL of Ea2Mab-7 (red line) followed by anti-mouse IgG conjugated with Alexa Fluor 488 treatment. Fluorescence data were collected using the SA3800 Cell Analyzer. Black line, control (no primary antibody treatment). Note that the expression of each Eph receptor was confirmed previously [31].

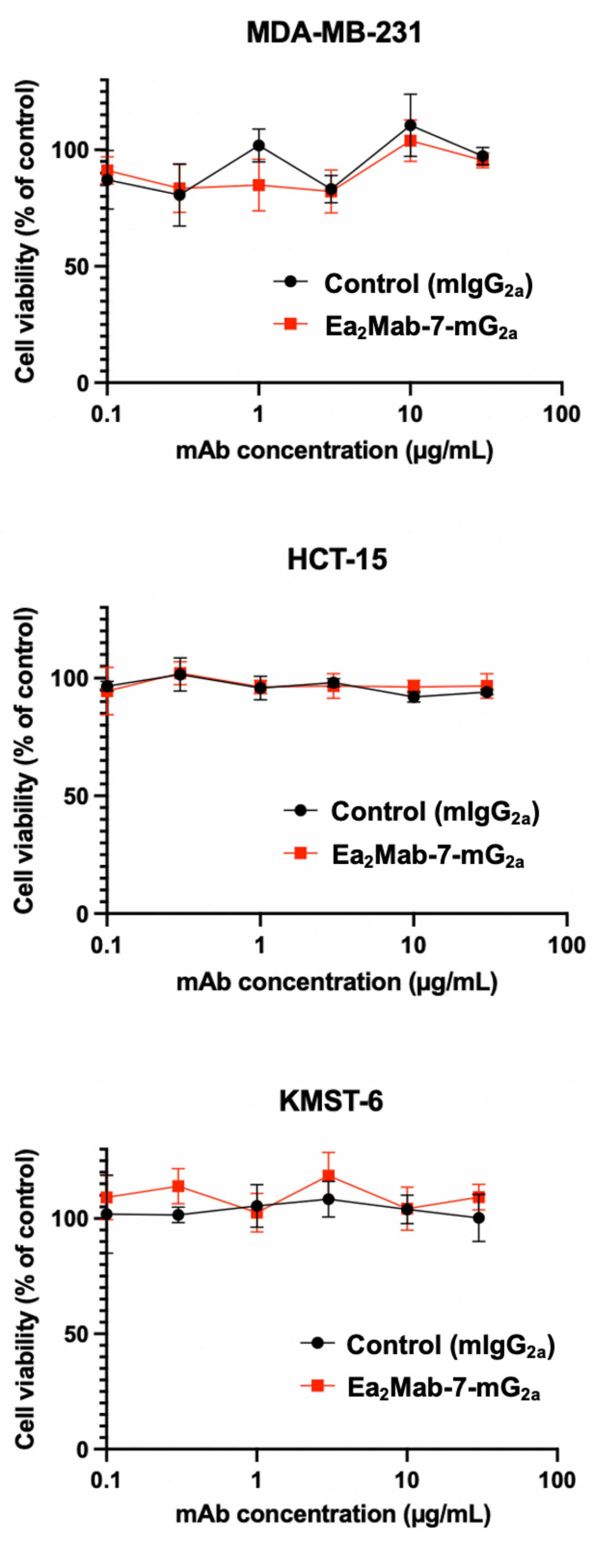

Supplementary figure S2: Effect of Ea$_2$Mab-7-mG$_{2a}$ on cell proliferation. MDA-MB-231, HCT-15, and KMST-6 were treated with an isotype control IgG$_{2a}$ mAb (PMab-231) or Ea$_2$Mab-7-mG$_{2a}$ at indicated concentration (0.1 ~ 30 μg/mL) for 6 days. The cells were treated with CellTiter-Glo®3D Cell Viability Assay reagent and the luminescence was measured using GloMax®. Cell viability (% of control) was determined.

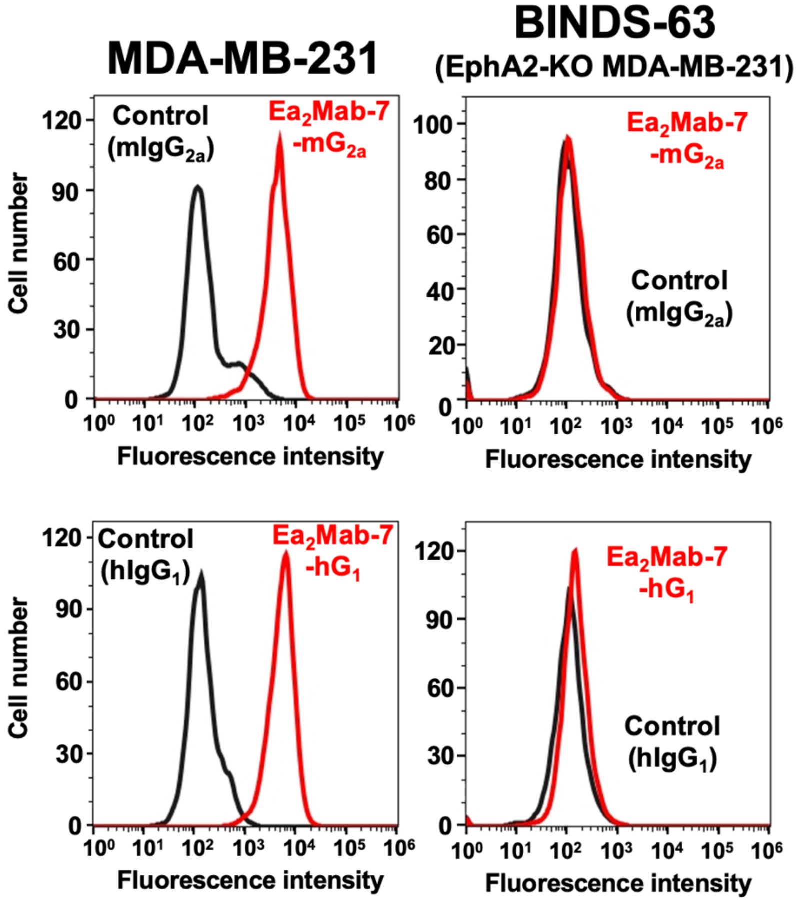

Supplementary figure S3: Flow cytometric analysis using EphA2KO-MDA-MB-231. MDA-MB-231 and BINDS-63 (EphA2KO-MDA-MB-231) were treated with 1 μg/mL of an isotype control IgG_{2a} mAb (PMab-231, black) or 1 μg/mL of Ea_2Mab-7-mG_{2a} (red). The mAbs-treated cells were incubated with anti-mouse IgG conjugated with Alexa Fluor 488. These cells were treated with 1 μg/mL of an isotype control human IgG_1 mAb (humCvMab-62, black) or 1 μg/mL of Ea_2Mab-7-hG_1 (red). The mAbs-treated cells were incubated with anti-human IgG conjugated with FITC. The fluorescence data were collected using the SA3800 Cell Analyzer.

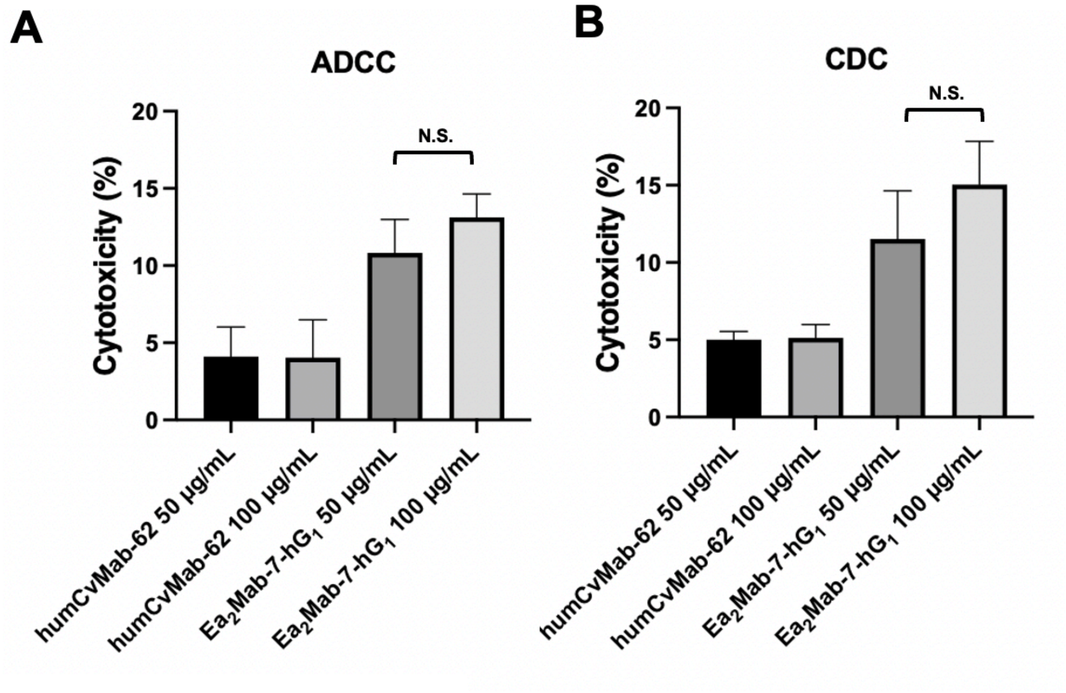

Supplementary figure S4: ADCC and CDC by Ea_2Mab-7-hG_1 against MDA-MB-231. The cells were incubated with effector splenocyte derived from BALB/c nude mice (A) or rabbit complement (B) in the presence of Ea_2Mab-7-hG_1 or control $hIgG_1$ (humCvMab-62). Calcein release into the medium was measured, and cytotoxicity was determined. Values are shown as the mean ± SEM (n = 3). N.S., not significant.

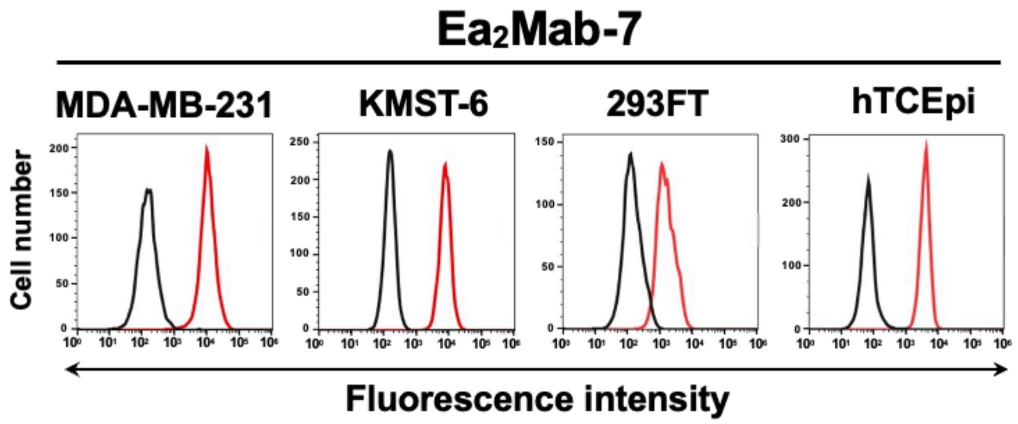

Supplementary figure S5: Flow cytometric analysis of Ea2Mab-7 in normal fibroblast and epithelial cell lines. MDA-MB-231 (positive control), embryonic fibroblasts KMST-6, embryonic kidney 293FT, and TERT-expressed normal cornea epithelial cell line hTCEpi were treated with 10 μg/mL of Ea2Mab-7 (red line) followed by anti-mouse IgG conjugated with Alexa Fluor 488 treatment. Fluorescence data were collected using the SA3800 Cell Analyzer. Black line, control (no primary antibody treatment).
